# Can Twitter Be a Source of Information on Allergy? Correlation of Pollen Counts with Tweets Reporting Symptoms of Allergic Rhinoconjunctivitis and Names of Antihistamine Drugs

**DOI:** 10.1371/journal.pone.0133706

**Published:** 2015-07-21

**Authors:** Francesco Gesualdo, Giovanni Stilo, Angelo D’Ambrosio, Emanuela Carloni, Elisabetta Pandolfi, Paola Velardi, Alessandro Fiocchi, Alberto E. Tozzi

**Affiliations:** 1 Multifactorial Disease and Complex Phenotype Research Area, Bambino Gesù Children’s Hospital IRCCS, Rome, Italy; 2 Department of Informatics, “Sapienza” University of Rome, Rome, Italy; University of Warwick, UNITED KINGDOM

## Abstract

Pollen forecasts are in use everywhere to inform therapeutic decisions for patients with allergic rhinoconjunctivitis (ARC). We exploited data derived from Twitter in order to identify tweets reporting a combination of symptoms consistent with a case definition of ARC and those reporting the name of an antihistamine drug. In order to increase the sensitivity of the system, we applied an algorithm aimed at automatically identifying jargon expressions related to medical terms. We compared weekly Twitter trends with National Allergy Bureau weekly pollen counts derived from US stations, and found a high correlation of the sum of the total pollen counts from each stations with tweets reporting ARC symptoms (Pearson’s correlation coefficient: 0.95) and with tweets reporting antihistamine drug names (Pearson’s correlation coefficient: 0.93). Longitude and latitude of the pollen stations affected the strength of the correlation. Twitter and other social networks may play a role in allergic disease surveillance and in signaling drug consumptions trends.

## Introduction

The Internet is increasingly exploited as a source of information on the population’s health.

Analysis of media reports [1], search engine queries [2], Wikipedia usage [3] and social networks provide data which may allow to assess and monitor the health status of a population in real time.

Twitter is a popular social network, based on the sharing of short messages of up to 140 characters. The potential power of this medium for public health is intrinsic to its nature: people tweet about their personal lives, and sometimes include in their messages information on their health status. The large number of Twitter users (271 million as of June 30^th^, 2014, generating over 500 million daily tweets, https://investor.twitterinc.com/releasedetail.cfm?releaseid=862505) allows to aggregate large amount of data and identify trends in disease prevalence.

On the basis of this observation, Twitter has mainly been used as a source of data on infectious diseases [4]. In particular, a number of studies showed a correlation between recurrence of influenza-related terms on Twitter and figures reported by traditional influenza surveillance systems [5–7].

Social networks and other Internet-based means of communication (eg. emails) have previously been investigated as potentially useful media for improving the care of patients affected with allergic diseases [8]. A study by Imonikhe et al. assessed information seeking behaviors of patients affected with allergic conjunctivitis [9]. Moreover, it has been recently shown that temporal variation in regional pollen counts correlates with Google searches for terms related to pollen allergy [10]. To our notice, Twitter has never been studied for allergic disease surveillance.

We conducted the present study with the aim of investigating the potential of Twitter as a source of information on allergic disease prevalence, on the basis of the observation that Twitter users affected with allergic rhinoconjunctivitis (ARC) may write tweets including combinations of specific symptoms or names of drugs commonly used to treat this condition. Our objective was to test the reliability of such tweet trends as a proxy for trends of ARC. To this aim, as no official surveillance data for ARC or other allergic diseases is available as a term of comparison, we took into account the correlation of clinical symptoms in allergic patients with aeroallergen level. We therefore investigated the correlation between US pollen counts obtained from the American Academy of Allergy, Asthma & Immunology (AAAAI) and trends of tweets, geolocalized in the US, reporting names of antihistamine drugs and symptoms of allergic rhinoconjunctivitis (ARC).

## Materials and Methods

### Twitter data

Starting from February 1^st^, 2013 and until September 30^th^, 2013, through the available application programmer’s interfaces (APIs, https://dev.twitter.com/docs/streaming-apis) we acquired a sample of the worldwide Twitter traffic, including at least one of 82 symptom-related terms. Such terms were selected as follows: first, we identified 17 singleton terms composing 4 queries based on case definitions (influenza-like illness, cold, gastroenteritis, allergy) adopted by the Influenzanet system (https://www.influenzanet.eu/en/results/?page=help#casedef). Secondarily, we applied to each of these terms an algorithm, which automatically detects naive English words related to specific medical concepts, as described elsewhere [11]. This algorithm allowed us to retrieve 65 additional jargon keywords. Therefore, we used a total of 17 technical keywords + 65 jargon keywords to acquire a sample of tweets which likely correspond to almost 100% of the Twitter traffic with those terms (being up to 1% of the total Twitter traffic). Due to network and hardware problems, it was not possible to collect the data on May 12^th^, and during the period between the 13^th^ and the 21^st^ of June, 2013.

In order to remove duplicates, avoid spam, and focus only on tweets from common users, we processed the data applying the following filters: remove all the copies of tweets appearing more than once in the collection; remove all those tweets that contained hyperlinks. Subsequently, we built a system that allows monitoring collected tweets and producing time series for the requested information, similarly to Google Trends (http://www.google.com/trends/). Our system shows the absolute frequency of terms and allows for complex Boolean queries.

We then used the geo-localization algorithm described elsewhere [11] to reliably consider the Twitter traffic originated in the US only. Specifically, the geo-localization criteria were as follows:

1. US GPS coordinates
2. explicit US place code
3. US-related time zone
4. place indicated in user’s profile included in those reported in http://en.wikipedia.org/wiki/List_of_U.S._states in the following fields: Common name, State Capital, Most populous city.

### Query development and evaluation

In order to extract from our dataset tweets actually reporting a complaint of ARC, we adapted the “Allergy” case definition adopted by the Influenzanet system (https://www.influenzanet.eu/en/results/?page=help#casedef) to a Boolean query: (allergy OR hay fever OR runny nose OR bloodshot eyes) AND (NOT (fever OR chills))

Each of the technical terms was expanded with the jargon terms identified by our algorithm.

For a more complete description of this method, see S1 Text.

On the same dataset, we extracted a series of tweets reporting the brand names of the most common antihistamine drugs, both by prescription and over the counter. The list of brand names, available in S2 Text, was obtained combining information from different websites (http://www.webmd.com/allergies/guide/allergy-medications; http://www.rxlist.com/allergy_medications-page6/drugs-condition.htm#medication; http://www.intelihealth.com/article/common-allergy-medications).

We used linguistic features (e.g. pronouns such as “I“, “my”, and other expressions of identity) to exclude tweets in which drugs were advertised rather than actually mentioned by a user.

Subsequently, from our dataset, we extracted 200 tweets matching the Allergy query and 200 tweets matching the antihistamine query. Tweets were examined independently by three of the Authors (EA, FG, AET), in order to test if the extracted tweets were consistent with the ARC case definition, or actually reported the use of an antihistamine drug. The tweet examination yielded a 15% false positive rate with a precision of 0.85 for the ARC, and a 3% false positive rate with a precision of 0.97 for the antihistamine tweets.

### Pollen counts and correlations

We obtained from the AAAAI the National Allergy Bureau (NAB) pollen counts from February 1^st^, 2013 and until September 30^th^, 2013 for 45 US stations, located in 29 States, as reported on S3 Text.

Each station reported daily data for single pollens and a total pollen count, with a variable percentage of missing data. Pollen data from San Juan (PR) were excluded from the analysis.

In order to compensate for daily variations, we calculated weekly pollen counts (average of pollen counts from Mondays to Sundays) and we took into account the following spatial levels:

- city
- state (average of the weekly city pollen counts from each State)
- climate region (average of the weekly city pollen counts from each climate region, see http://www.ncdc.noaa.gov/monitoring-references/maps/us-climate-regions.php)
- all US (sum and average of the weekly city pollen counts)

We calculated the pairwise Pearson’s correlation coefficients between each weekly pollen count series and the following weekly tweet trends (obtained from an average of daily tweets from Mondays to Sundays):

- ARC tweets geolocalized in all US (ARC-US)
- antihistamine tweets geolocalized in all US (AH-US)

Incomplete time-matched values were excluded.

We performed a multivariable regression analysis in order to study which factors affected the Pearson’s correlation coefficients between each pollen station and the tweet trends. We built the models with the Pearson’s correlation coefficient (between each pollen station and the tweet trends) as dependent variables, and the following independent variables: latitude (tens of degrees, centered on the mean), longitude (tens of degrees, centered on the mean), state population density (100 inhabs/sqkm), pollen count (hundreds of units) and pollen data completeness (proportion of weeks with at least one pollen recording). As we noticed a non linear effect of latitude on the correlation, we included a quadratic term for the latitude in the analysis.

The software R ver. 3.1.2 was used for the analysis.

## Results

For the selected period, we obtained a total of 43,467 tweets consistent with the ARC case definition (see S1 Dataset), and a total of 17,987 tweets consistent with the antihistamine query (see S2 Dataset).

In the ARC-US, unique users were 40,308, and median number of tweets/user was 1.076 (range 1–18). In the AH-US, unique users were 16,767, and median number of tweets/user was 1.073 (range 1–9).

**Fig 1:**
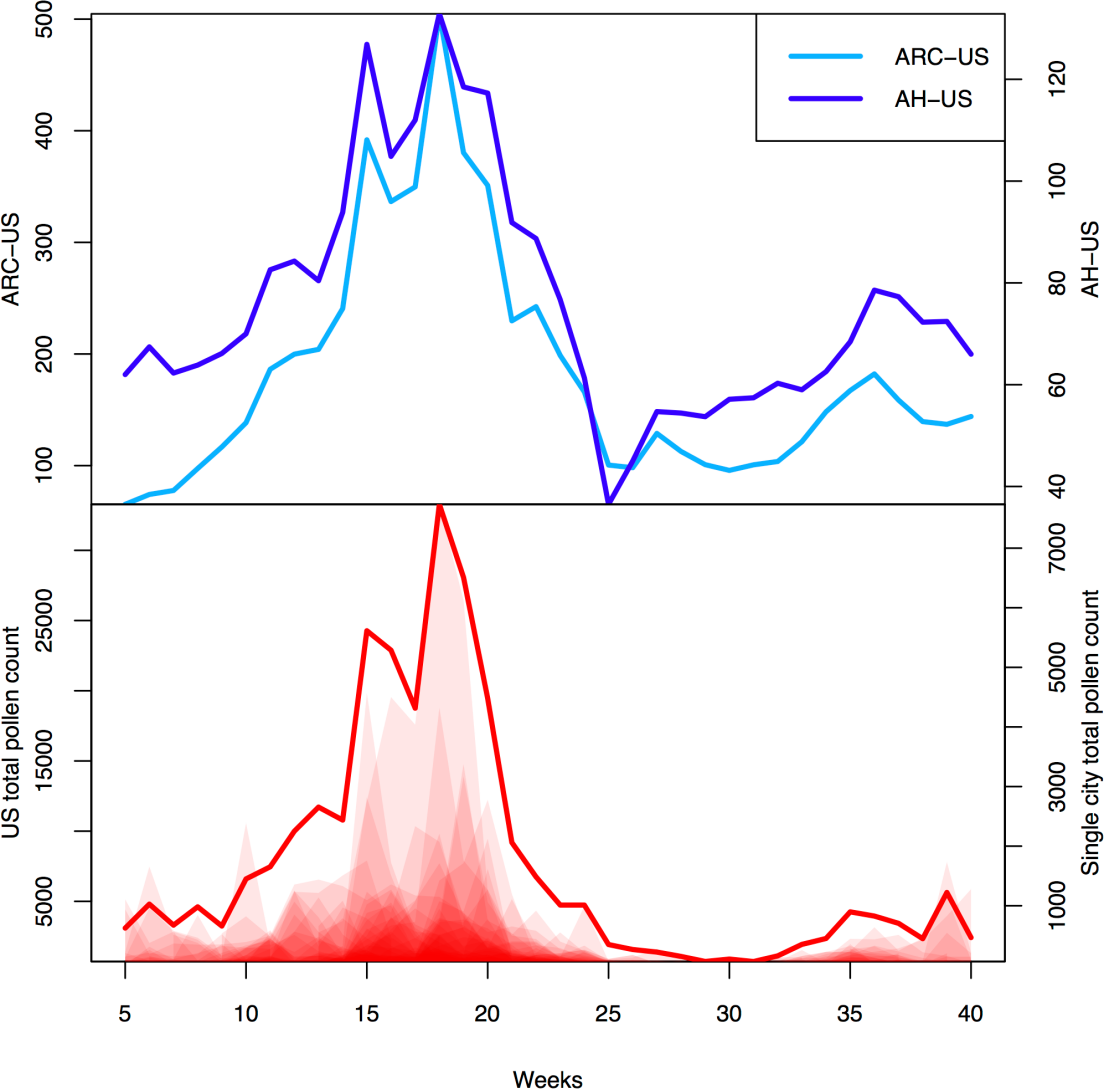
Tweet trend for ARC case definition, tweet trend for antihistamine drugs and US total pollen count. In the upper plot: the light blue line represents the weekly averages of tweets satisfying the ARC case definition; the dark blue line represents the weekly averages of tweets including the name of an antihistamine drug. Tweet trends are obtained from tweets which were geolocalized in the US and are plotted on different scales to emphasize the similarities of the trends. In the lower plot: the red solid line represents the sum of the total pollen counts obtained from all US stations; the shaded red areas represent the single city total pollen counts. The single city total pollen counts are represented on the same scales. The sum of the total pollen counts is plotted on a specific scale to allow trend comparison with the single city pollen counts. All trends represent weekly averages for the 35-week (242 day) period starting in week 5 (January 2013) through week 40 (September 2013).

Fig 1 shows the trend of the following series: all US total pollen counts, ARC-US and AH-US.

S1 Fig shows the total pollen count trend for each climate area, state and city (each graph reporting also the ARC-US and the AH-US series for a straightforward visual comparison).

The sum of the city total pollen counts (all US) and both the tweet series showed a trimodal distribution, first peaking in early spring, secondarily in late spring and thirdly in early autumn.

The US total pollen count started to increase in early March (week 10), first peaked in the first half of April (week 15) and subsequently in mid May (week 18). Pollen counts progressively decreased reaching their minimum in the second half of July (week 29), and increased again in mid August (week 32), reaching two peaks in September (week 36 and week 39).

An increase in twitter-defined ARC started in late February (week 8), first peaked in the second half of April (week 15) and subsequently in mid May (week 18), then it decreased slowly throughout May and June, reaching its minimum in mid June (week 25). ARC tweets raised again in mid August (week 32), peaking in the first week of September (week 36).

Antihistamines were reported from week 9, the trend first peaked in the second half of April (week 15) and subsequently in mid May (week 18), reaching its minimum already in early June (week 26). A further rise in antihistamines was recorded from week 26, with a peak in early September (week 35).

The level of correlation between the ARC-US and the AH-US was high (0.95).

The ARC-US had correlation coefficient of 0.95 with the sum and of 0.89 with the average of the total pollen counts of all cities; the AH-US had correlation coefficient of 0.93 with the sum and of 0.91 with the average of the total pollen counts of all cities.

**Fig 2:**
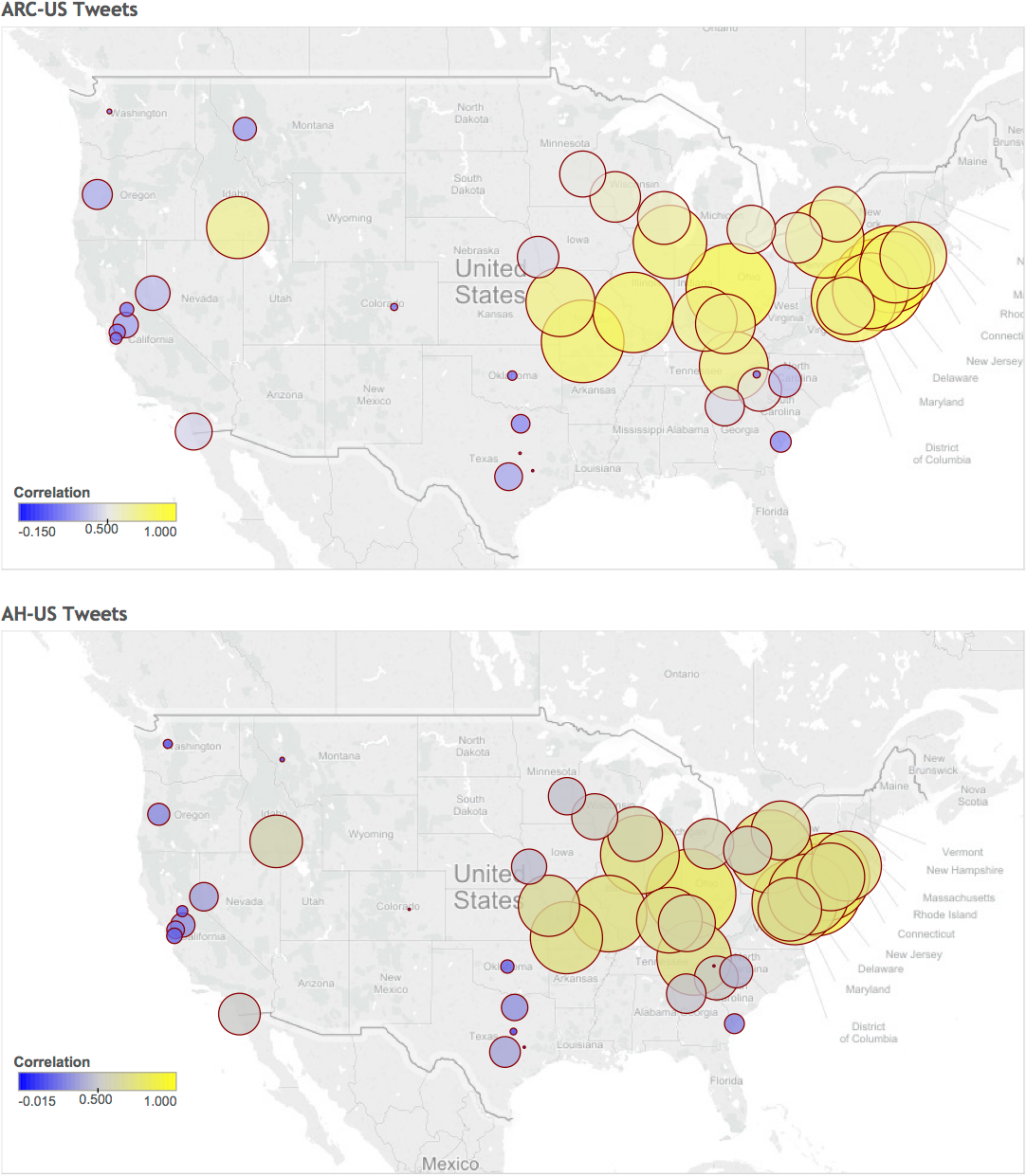
Map of correlations between tweets and pollen series. The map shows circles representing the level of correlation between total pollen counts for each city and ARC-US tweet series (above) and AH-US tweet series (below). The strength of the correlation is represented both by the size and by the color of the circles. Colors from blue to yellow represent increasing correlation.

Table 1 shows the correlation of the ARC-US series and of the AH-US series with each pollen count by city, state and climate area. The maps in Fig 2 offer a visual presentation of the correlations of each city with ARC-US and AH-US.

**Table 1:** Correlation of the ARC-US series and of the AH-US series with each pollen count by city, state and climate area.

****Climate Area****	****State****	****City****	****ARC-US****	****AH-US****
central			0,892	0,811
	IL		0,776	0,714
		Melrose Park	0,776	0,714
	KY		0,681	0,651
		Lexington	0,7	0,616
		Louisville	0,716	0,683
	MO		0,85	0,754
		Kansas City	0,735	0,653
		Springfield	0,803	0,698
		St.Louis	0,801	0,711
	OH		0,884	0,8
		Dayton	0,884	0,8
	TN		0,735	0,7
		Knoxville	0,735	0,7
east north central			0,562	0,541
	MI		0,569	0,575
		St.Claire Shores	0,569	0,575
	MN		0,505	0,478
		Minneapolis	0,505	0,478
	WI		0,584	0,549
		LaCrosse.Onalaska	0,572	0,533
		Waukesha	0,591	0,583
northeast			0,906	0,831
	CT		0,718	0,697
		Waterbury	0,718	0,697
	DC		0,699	0,665
		Washington	0,699	0,665
	DE		0,788	0,72
		New.Castle	0,788	0,72
	MD		0,816	0,743
		Baltimore	0,816	0,743
	NJ		0,877	0,784
		Springfield	0,877	0,784
	NY		0,807	0,751
		New York	0,756	0,69
		Olean	0,8	0,731
		Rochester	0,647	0,637
	PA		0,631	0,594
		Erie	0,572	0,548
		Philadelphia	0,927	0,856
northwest			0,307	0,304
	ID		0,702	0,578
		Twin Falls	0,702	0,578
	OR		0,317	0,307
		Eugene	0,317	0,307
	WA		0,116	0,169
		Seattle	0,116	0,169
south			0,254	0,405
	OK		0,134	0,211
		Oklahoma City	0,134	0,211
	TX		0,258	0,404
		Austin	0,292	0,384
		College.Station	-0,149	0,0255
		Flower Mound	0,196	0,331
		Waco 1	-0,0337	0,129
		Waco 2	0,613	0,679
southeast			0,496	0,517
	GA		0,459	0,487
		Marietta	0,461	0,487
		Savannah	0,201	0,3
	NC		0,132	0,16
		Asheville	0,121	0,0717
		Charlote	0,36	0,442
	SC		0,496	0,515
		Greenville	0,496	0,515
southwest			0,121	0,0717
	CO		0,121	0,0717
		Colorado Springs	0,121	0,0717
west			0,27	0,34
	CA		0,223	0,297
		Pleasanton	0,181	0,275
		Roseville	0,155	0,195
		San Diego	0,428	0,506
		San Jose	0,154	0,215
		Stockton	0,283	0,326
	NV		0,367	0,38
		Reno.Sparks	0,367	0,38
west north central			0,449	0,448
	MT		0,257	0,11
		Missoula	0,257	0,11
	NE		0,464	0,463
		Omaha	0,464	0,463

The highest correlation was reported between the ARC-US and the AH-US series and cities located in the northeast (0.9 and 0.83) and central (0.89 and 0.81) climate areas: Philadelphia, PA (0.93 and 0.85), Dayton, OH (0.88 and 0.8) and Springfield, NJ (0.87 and 0.78).

The multivariable regression analysis showed that the level of correlation between the city pollen counts and the ARC-US series was affected by longitude (coeff. 0.09, 95% CI 0.05–0.14) and latitude (coeff. 0.24, 95% CI 0.1–0.38; quadratic term: coeff -0.4, 95% CI -0.68 to -0.13). On the other hand, the correlation between the city pollen counts and the AH-US series was affected by longitude (coeff. 0.08, 95% CI 0.04–0.12) and latitude (coeff. 0.12, 95% CI -0.002 to 0.025; quadratic term: coeff -0.3, 95% CI -0.54 to -0.06). Pollen levels, completeness of pollen data and state population density did not significantly affect the Pearson’s correlation coefficients.

## Discussion

Our study shows a high correlation between US pollen counts and tweets reporting a complaint of ARC. We also show a high correlation between pollen counts and tweets reporting names of antihistamine drugs. Our data also show that the tweet series exactly parallel the three peaks of atmospheric pollen spread of early spring (tree pollens), spring (grass pollen) and late summer (ragweed).The trends of ARC and antihistamine tweets in early June suggest that the time of maximum subjective perception of pollinosis is the start of the spring season. Early pollens, released mainly in March-June, are related to ARC complaints in Twitter users more than pollens that are present in the US in late summer. Yet, during August, the tweets indicate an increase of interest in antihistamines. Taken together, the findings seem to indicate that the spring pollens (grass and tree pollens) are more associated with antihistamine use than ragweed. If so, the common wisdom that weeds are the major responsible of allergic rhinitis in the US (http://www.niaid.nih.gov/topics/allergicDiseases/Documents/PollenAllergyFactSheet.pdf) should be reconsidered.

Moreover, the drop of tweets on antihistamines in early June can be interpreted in two ways. Either patients are less interested in speaking about their medication, but continue to take it up to the end of the pollen season; or patients stop the treatment too early [12].

The pollen counts from the different stations reflected heterogeneous environments, with different pollen seasons. We identified a high correlation of the tweet series with pollen counts from stations located in eastern and central areas. Moreover, the multivariable analysis showed that the level of correlation was influenced by longitude and latitude. This might be explained by the fact that most tweets are written by people located in the US eastern regions (http://firstmonday.org/article/view/4366/3654).) As pointed out in a previous study on influenza symptoms [6], we may speculate that the sensitivity of our system was increased by the inclusion of naive terms as automatically identified by our algorithm. Moreover, its specificity may be increased by the choice of using a combination of symptoms defining a syndrome, rather than single keywords.

The Internet has previously been studied as a source of information on prescription drugs. A correlation between search volumes and the utilization rates of seasonal prescription drugs has been demonstrated [13]. The use of prescription drugs among college students [14], and adverse drug reactions [15] have been investigated on Twitter. To our knowledge, no web-based study has previously focused on antihistamines. Of course, antihistamines are used as a treatment for a number of diseases. Among these diseases, ARC is probably the one with the highest seasonality. Based on this observation, we may hypothesize that false positives (i.e. users reporting a name of an antihistamine drug but not saying he or she used it) or users reporting the use of an antihistamine for the treatment of diseases other than ARC may represent a sort of constant “background noise”, which should not bias the seasonal trend of antihistamine use for ARC treatment. Therefore, the trend of the recurrence of antihistamine names on Twitter may represent a proxy of ARC prevalence.

Our study has a number of limitations.

We acquired our dataset selecting tweets which included at least one of 82 symptom-related keywords, as described in the methods and in [11]. This implies two biases affecting the system’s sensitivity. First, we might have missed a number of tweets which actually described an ARC but which were not detectable through our keywords. Secondarily, taking into account the dataset’s nature, the AH-US series included tweets with the name of an antihistamine drug, selected among tweets with at least one symptom. Therefore, a number of tweets including the name of an antihistamine drug, but not including any symptom, might have been missed.

Moreover, the tweet series were geolocalized using a “conservative” algorithm, which uses GPS coordinates and a combination of additional information in the user’s tweets [11]. The development of more effective geolocalization techniques, taking also into account the networks of users (e.g. their social relations) would allow gathering finer spatial information, thus increasing the soundness of the system. This is an on-going research theme.

Finally, our data refer to a unique season. In order to validate our findings, trends should be evaluated on more than one season.

Despite these biases, our results represent a proof of concept of the potential role of social networks in signaling allergic symptoms and drug consumption trends.

The epidemiology of ARC is scarcely known: most prevalence data are derived from phone interviews [16,17], and no system is available for allergic disease surveillance. An implemented social network-based symptom-detection system may represent a step towards a clearer epidemiologic characterization of this disease, and may clarify the real clinical impact of different kinds of pollen. Moreover, our findings may have a wide range of potential applications. As a matter of fact, the interest for integrated care pathways for airway diseases is under the look of a series of international organization, including GARD [18]. A model including specific pollen counts, other environmental data as weather and pollen forecasts, Google search data and a geolocalized series of tweets reporting ARC symptoms and antihistamine names could constitute the basis of an alert system, which may represent a decision support system for drug intake and possible avoidance of specific locations, informing allergic patients on areas where the risk of developing allergic symptoms is higher.

## Supporting Information

S1 Text Tracing allergy on Twitter. (DOCX)

S2 Text Brand names of antihistamine drugs. (DOCX)

S3 Text NAB Station Data Attribution List. (PDF)

S1 Dataset ARC tweets. (GZ)

S2 Dataset AH tweets. (GZ)

S1 Fig Total pollen counts by climate area, state and city. Each graph displays the total pollen count trend for each climate area, state and city. For comparison purposes, in each graph we show the ARC-US and the AH-US tweet trend.
(PDF)
