# Supplementary material for: Can Twitter Be a Source of Information on Allergy? Correlation of Pollen Counts with Tweets Reporting Symptoms of Allergic Rhinoconjunctivitis and Names of Antihistamine Drugs
Source: PLoS One. 2015 Jul 21;10(7):e0133706. doi: 10.1371/journal.pone.0133706 (PMC4510127; doi:10.1371/journal.pone.0133706)
Supplement: S1 Text — (DOCX) [file pone.0133706.s001.docx]

***Tracing Allergy on Twitter***

Twitter mining algorithms used in previous health-related studies have measured the occurrence of single pre-specified terms, consisting of either the name or synonyms of a clinical condition (eg: *H1N1* or *swine flu*) or of words, arbitrarily chosen by the authors, related to the clinical syndrome itself (eg. *flu, vaccine, tamiflu*) and/or to specific expression, e.g. fear of infection, as in [1]*.* However, this kind of approach may suffer from major biases, which we will illustrate with an example. Consider the following striking difference in the usage of terms describing the same health conditions, the first by a clinician, the second by a patient: “*Allergic rhinitis is an inflammation of the nasal membranes that is characterized by sneezing, nasal congestion, nasal itching, and rhinorrhea, in any combination*”[[1]](#footnote-1) “*l suffer from a blocked nose like lm going to get a cold but it dont happen, My eyes water esp as day goes on and this year my chest really aches (upper chest)*”[[2]](#footnote-2). Here, the similarity between these symptom descriptions is not so obvious as to allow capture by an automated system, for two reasons: First, in blogs and forums, people are motivated by a communication need (frequently “one-to-one”, between just two individuals), rather than by an information need, and therefore naïve language is often preferred to technical language. Thus, being able to understand the way people talk about medical terms (diseases, symptoms, and treatments) in “peer to peer” communications is crucial for an effective monitoring of health-related behaviors based on social data. Second, it is likely that, in their tweets, most users will describe a combination of symptoms rather than a diagnosis. An approach that takes into account only disease-related keywords can miss a large volume of messages in which users include a mix of signs and symptoms that may in reality be describing a clinical syndrome. With reference to the previous example, high co-occurrence rates of symptoms like *blocked nose, congested chest, eyes* *water* and others, may be used to trigger an alarm in syndromic surveillance systems.

To cope with these issues, we adopted an entirely different approach. We first developed an algorithm to automatically learn a variety of expressions that people use to describe their health conditions, thus improving our ability to detect health-related “concepts” expressed in non-medical terms and, in the end, producing a larger body of evidence. We then implemented a Twitter monitoring instrument to finely analyze the presence and combinations of symptoms in tweets. We transformed syndrome definitions into a Boolean query, thereby basing our analysis on a combination of symptoms (each expanded with a set of correspondent naïve terms) rather than on a suspected or final diagnosis.

Concerning ALLERGY, we started from the definition adopted by the official definition adopted by the Influenzanet system (<https://www.influenzanet.eu/en/results/?page=help#casedef>):

- Not ILI^ECDC^
- Not fever or chills or Temperature ≥38C
- At least 1 of: Runny or blocked nose; sneezing; watery, bloodshot eyes

We created a Boolean query with positive and negative (Not ILI^ECDC^, Not fever, Not chills) conditions, and we expanded every query term with all its synonyms and near-synonyms detected expressions.

The negative condition Not ILI^ECDC^ is represented by the Boolean expression:

1. *[(fever)**(chills))*  *(malaise)*  *(headache)*  *(myalgia)]*  *[(cough)*  *(pharyngitis)*  *(dyspnea)]*

while the positive conditions are represented by:

1. *[(runny nose)**(blocked nose)*  *(sneezing)*  *(watery eyes)*  *(bloodshot eyes)* *(allergy)]*

The complete query is then:

*(3) NOT(1)*  *NOT(fever)*  *NOT(chills)*  *(2)*

This query is extended replacing every term with the disjunction of its correspondent alternate terms retrieved by our algorithm, for example: *watery, bloodshot eyes*🡪 *watery eyes, bloodshot eyes, inflamed eyes, inflammation of eyes, eye water, eye redness, itchy eyes, eye itch, stinging eye, sick eye, irritated eye, puffy eye, burning eye, swollen eye, teary eye.* Query expansion with naïve terms considerably increases the number of matches, thus providing a statistically reliable body of evidence.

Examples of tweets matching the ALLERGY query are:

“*Allergic Rhinitis, why are you making my life worse? Is this a curse?! “*

*“cant take this watery eyes, sniffling and sneezing #allergies”*

*“Sneezing, runny nose and itchy eyes? Hayfever whyyyy”*

*“Itchy eyes, twitchy nose, sneezing. I hate you, hayfever”*

For the purpose of evaluation, we also selected a number of tweets not matching the query but including at least one of the symptoms of the query. Examples are:

*“Ear infection, runny nose, sneezing, coughing, and most of all headaches. :/”*

*“This cough is slowly killing me. And this runny nose. And this sneeze that just won't happen”*

Furthermore, we geo-localize our matching tweets using a variety of methods, not described here for sake of space (the interested reader is referred to [2]). Therefore, we can produce a reliable estimate of Allergy cases in U.S. and even more fine-grained geographical distributions, for selected regions.

Detection of naïve language and symptom-driven keyword analysis (rather than disease-driven) represent a major difference with previous methods for syndromic surveillance. First, knowledge of naïve language provides a considerably larger corpus of evidence. Then, second, knowledge of patients’ language allows fine-grained queries to be performed on the Twitter corpus, separating, for example, patients with simple conjunctivitis or cold symptoms from those with an allergy, or a “true” ILI, thus solving a “noise” problem of web-based epidemiological data, pointed out in [1]. Third, our methodology (similarly to [1]) is very reliable in selecting only tweets of people that actually complain an allergy, rather than people worried by the possibility of being infected. In fact, people may say “*I hope I’ll never get an allergy*” but they are unlikely to say “*I’m afraid to get itchy eyes, twitchy nose and sneezing!*”.

1. Alex Lamb, Michael J. Paul, Mark Dredze (2013). *Separating Fact from Fear: Tracking Flu Infections on Twitter*. (NAACL), 2013.
2. P. Velardi, G. Stilo, E. Tozzi and F. Gesualdo Twitter mining for fine-grained syndromic surveillance, Artificial Intelligence in Medicine, Elsevier, AIIM-D-13-00148R2, in press (2014)
3. [Shaman J](http://www.ncbi.nlm.nih.gov/pubmed?term=Shaman%20J%5BAuthor%5D&cauthor=true&cauthor_uid=24302074), [Karspeck A](http://www.ncbi.nlm.nih.gov/pubmed?term=Karspeck%20A%5BAuthor%5D&cauthor=true&cauthor_uid=24302074), [Yang W](http://www.ncbi.nlm.nih.gov/pubmed?term=Yang%20W%5BAuthor%5D&cauthor=true&cauthor_uid=24302074), [Tamerius J](http://www.ncbi.nlm.nih.gov/pubmed?term=Tamerius%20J%5BAuthor%5D&cauthor=true&cauthor_uid=24302074), [Lipsitch M](http://www.ncbi.nlm.nih.gov/pubmed?term=Lipsitch%20M%5BAuthor%5D&cauthor=true&cauthor_uid=24302074). *Real-time influenza forecasts during the 2012-2013 season*. Nature Communications, 2013 Dec 3;4:2837. doi: 10.1038/ncomms3837.
4. Cleveland WS, Devlin SJ, Grosse E (1988) *Regression by local fitting: Methods, properties, and computational algorithms.* J Econ 37: 87-114. doi: 10.1016/0304-4076(88)90077-2

1. *http://emedicine.medscape.com/article/134825-overview* [↑](#footnote-ref-1)
2. *http://www.patient.co.uk/forums/discuss/* [↑](#footnote-ref-2)
