# Supplementary material for: Can Twitter Be a Source of Information on Allergy? Correlation of Pollen Counts with Tweets Reporting Symptoms of Allergic Rhinoconjunctivitis and Names of Antihistamine Drugs
Source: PLoS One. 2015 Jul 21;10(7):e0133706. doi: 10.1371/journal.pone.0133706 (PMC4510127; doi:10.1371/journal.pone.0133706)
Supplement: S2 Text — (DOCX) [file pone.0133706.s002.docx]

**Brand names of antihistamine drugs**

Aerius

Alavert

Allegra

Aller-Ease

Alzene

Astelin

Astepro

Azelastine

Azep

Benadryl

Cetirizine

Claratyne

Clarinex

Claritin

Clear-Atadine

Dimetapp

Fexal

Fexo

Fexorelief

Fexotabs

Kestine

Lorano

Lorapaed

Loratadine

Mucinex

Optivar

Patanase

Reditab

Seldane

Tavist

Telfast

Wal-itin

Xergic

Xyzal

Zyrtec
