# Supplementary material for: Can Twitter Be a Source of Information on Allergy? Correlation of Pollen Counts with Tweets Reporting Symptoms of Allergic Rhinoconjunctivitis and Names of Antihistamine Drugs
Source: PLoS One. 2015 Jul 21;10(7):e0133706. doi: 10.1371/journal.pone.0133706 (PMC4510127; doi:10.1371/journal.pone.0133706)
Supplement: S3 Text — (PDF) [file pone.0133706.s003.pdf]

NAB Station Data Attribution List
"Syndromic surveillance of allergy through social media"
Alberto E. Tozzi, MD
Bambino Gesù Ospedale Pediatrico

Guy Robinson, PhD
The Louis Calder Center
Armonk, NY

David Cypcar, MD FAAAAI
Allergy Partners of Western North Carolina
Asheville, NC

Jonathon Matz, MD FAAAAI &
David Golden, MD FAAAAI
Dr. Golden and Dr. Matz, LLC
Baltimore, MD

Linda B. Ford, MD FAAAAI
The Asthma and Allergy Center, PC
Bellevue (Omaha), NE

John T. Klimas, MD FAAAAI
Carolina Asthma and Allergy Center
Charlotte, NC

David R. Weldon, MD FAAAAI, FACAAI
Scott & White Clinic
College Station, TX

Robert A. Nathan, MD FAAAAI &
Daniel F. Soteres, MD MPH FAAAAI
Asthma & Allergy Associates, PC
Colorado Springs (Station 1), CO

William Storms, MD FAAAAI
The William Storms Allergy Clinic
Colorado Springs (Station 2), CO

Andy Roth
RAPCA
Dayton, OH

Philip E. Gallagher, MD FAAAAI
Allergy & Asthma Associates of Northeastern PA
Erie, PA

Kraig W. Jacobson, MD FAAAAI
Allergy & Asthma Research Group
Eugene, OR

Marie H Fitzgerald, MD
North Texas Pollen Station
Flower Mound, TX

Neil L Kao MD FAAAAI
Allergic Disease and Asthma Center
Greenville, SC

Jay Portnoy, MD FAAAAI
Children's Mercy Hospital
Kansas City, MO

Michael Miller, MD FAAAAI
Allergy, Asthma and Immunology
Knoxville (Station 1), TN

Beth Miller, MD
University of Kentucky Asthma Allergy & Immunology
Lexington (Station 1), KY

James L. Sublett, MD
FAAAAI Family Allergy & Asthma
Louisville (Station No 2), KY

Stanley M. Fineman, MD FAAAAI
Atlanta Allergy and Asthma Clinic
Marietta, GA

Joseph G. Leija, MD FAAAAI
Dr. Joseph Leija
Melrose Park, IL

Harold B. Kaiser, MD FAAAAI
Clinical Research Institute
Minneapolis, MN

NAB Station Data Attribution List
"Syndromic surveillance of allergy through social media"
Alberto E. Tozzi, MD
Bambino Gesù Ospedale Pediatrico

Emily Weiler
U of Montana, Ctr for Environmental Health Sciences
Missoula, MT

Michael McDowell
Division of Air Quality, DNREC, State of Delaware
New Castle, DE

Guy Robinson, PhD
Fordham College at Lincoln Center
New York, NY

Warren V. Filley, MD FAAAAI
OK Allergy Asthma Clinic, Inc.
Oklahoma City (Station No I), OK

Fred Lewis, MD FAAAAI
Fred Lewis, MD FAAAAI
Olean, NY

David Morris, MD
Allergy Associates of LaCrosse
Onalaska, WI

Donald J. Dvorin, MD FAAAAI
Allergic Disease Associates, P.C.
Philadelphia (Station No 1), PA

Allyson Tevirzian, MD
Allergy Medical Group of the Bay Area
Pleasanton, CA

Donald W. Pulver, MD FAAAAI
Allergy, Asthma & Immunology of Rochester
Rochester, NY

Sunil P. Perera, MD FAAAAI
Allergy Medical Group of the North Area
Sacramento, (Roseville) CA

Robert T. Reid, MD
Erik and Ese Banck Clinical Research Center
San Diego (Station 1), CA

Robert T. Reid, MD
Robert T. Reid, MD FAAAAI
San Diego (Station 2), CA

Theodore Chu, MD FAAAAI
Theodore Chu, MD FAAAAI
San Jose (Station No 1), CA

Alan Goldsobel, MD FAAAAI &
James Wolfe, MD FAAAAI
Allergy and Asthma Associates of No. California
San Jose (Station No 2), CA

Benjamin Bolanos, PhD
Medical Sciences Campus, UPR
San Juan, PR

Brad H. Goodman, MD &
Bruce D. Finkel, MD
Coastal Allergy & Asthma, P.C.
Savannah, GA

Frank Virant, MD FAAAAI
Northwest Asthma & Allergy Center
Seattle, WA

Leonard Shapiro, MD FAAAAI
Allergy & Asthma Associates
Sparks, NY

Rhizza Adams
Springfield -Greene County Health Department
Springfield, MO

Leonard Bielory, MD FAAAAI
STARx Allergy & Asthma Center
Springfield, NJ

NAB Station Data Attribution List
"Syndromic surveillance of allergy through social media"
Alberto E. Tozzi, MD
Bambino Gesù Ospedale Pediatrico

Andrew I. Dzul, MD
Lakeshore Ear Nose & Throat Center
St. Clair Shores, MI

Wayne Wilhelm
St. Louis County Health Department
St. Louis, MO

Gregory W. Bensch, MD FAAAAI &
George W Bensch, MD FAAAAI
Allergy, Immunology and Asthma Medical Group
Stockton, CA

Richard Henry, MD
Asthma & Allergy of Idaho
Twin Falls, ID

N.J. Amar, MD FAAAAI
Allergy and Asthma Center
Waco (Station No 1), TX

Pramila K. Daftary, MD FAAAAI
Allergy & Asthma Care of Waco
Waco (Station No 2), TX

Susan E. Kosisky MA
Walter Reed Army Medical Ctr.
Washington, DC

Christopher Randolph, MD
Waterbury, CT

Walter Brummund, MD, PhD, FAAAAI
Allergy & Asthma Centers, S.C.
Waukesha, WI
