# Supplementary material for: Can Twitter Be a Source of Information on Allergy? Correlation of Pollen Counts with Tweets Reporting Symptoms of Allergic Rhinoconjunctivitis and Names of Antihistamine Drugs
Source: PLoS One. 2015 Jul 21;10(7):e0133706. doi: 10.1371/journal.pone.0133706 (PMC4510127; doi:10.1371/journal.pone.0133706)
Supplement: S1 Fig — Each graph displays the total pollen count trend for each climate area, state and city. For comparison purposes, in each graph we show the ARC-US and the AH-US tweet trend. (PDF) [file pone.0133706.s006.pdf]

Pollen count trend for all climate areas

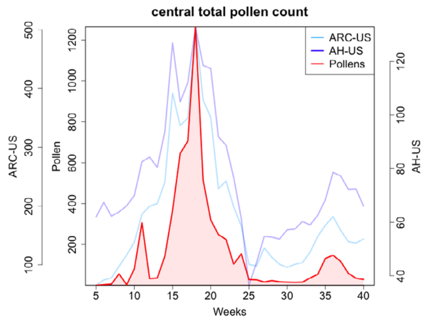

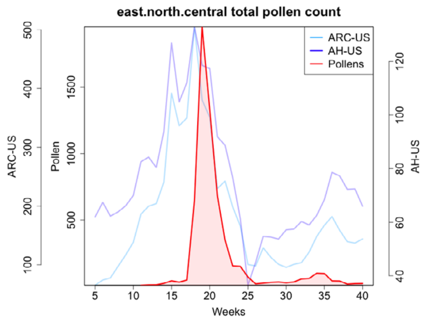

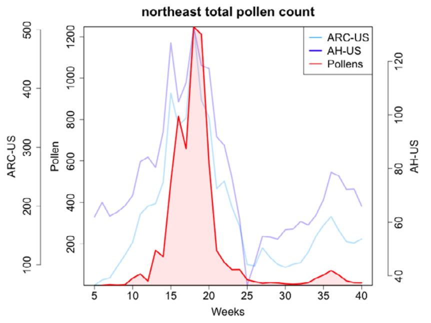

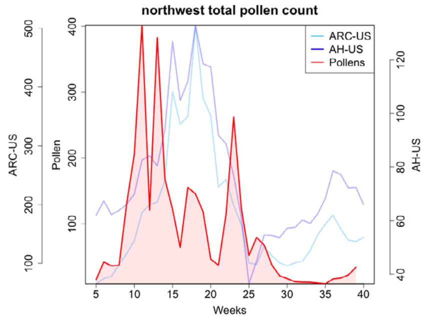

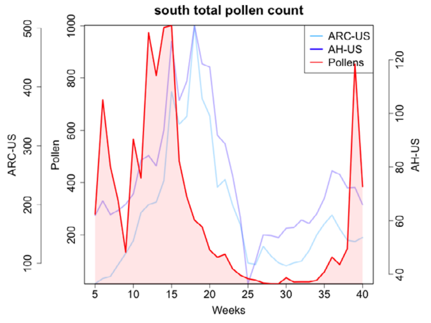

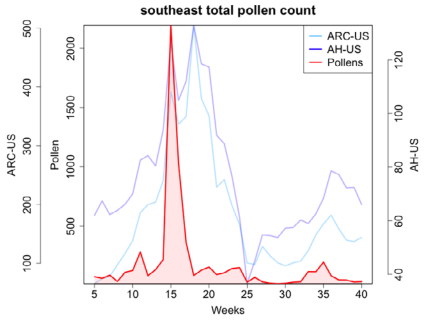

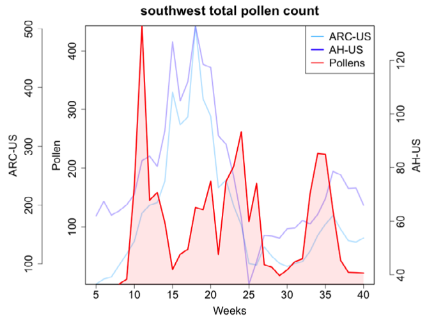

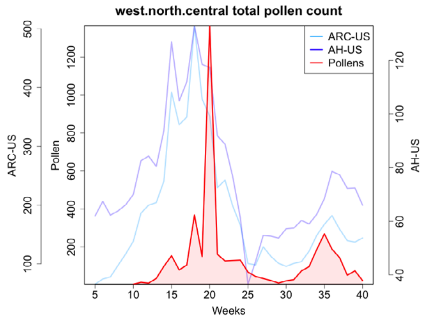

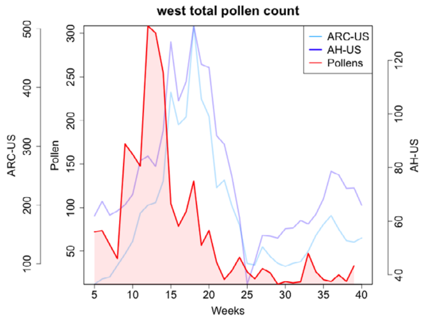

Pollen count trend for all states

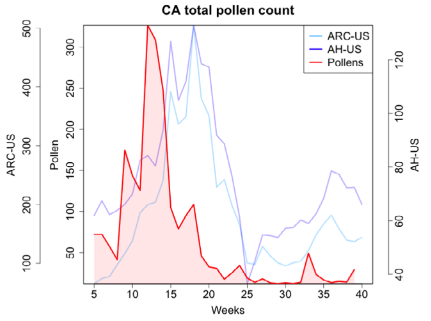

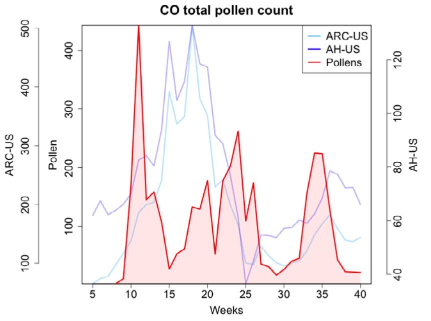

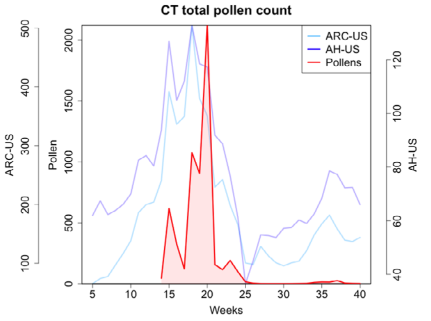

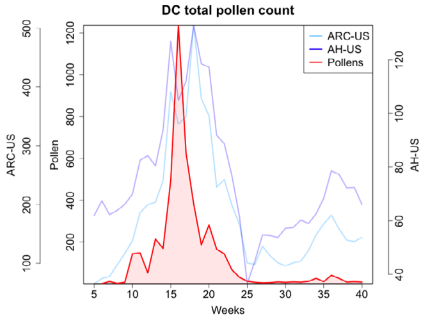

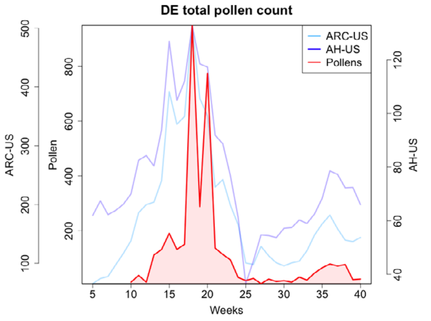

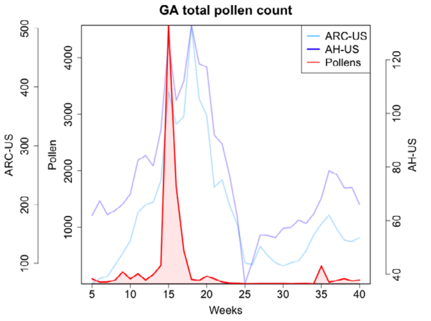

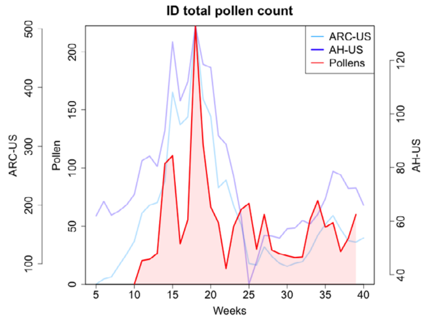

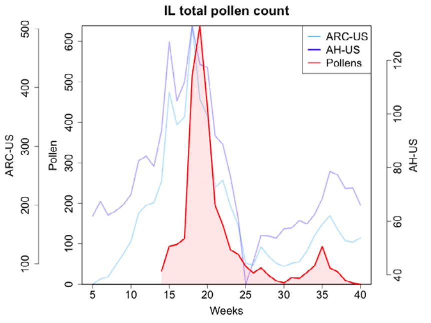

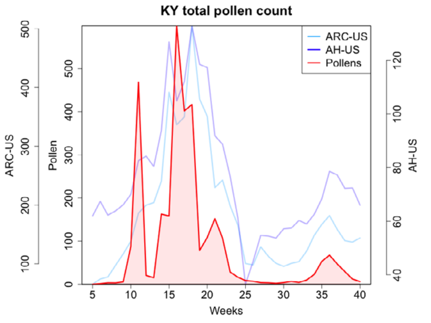

MD total pollen count

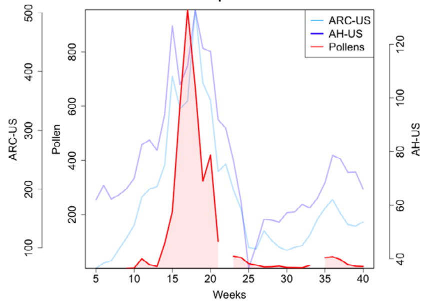

MI total pollen count

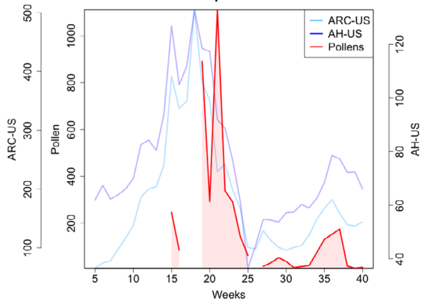

MN total pollen count

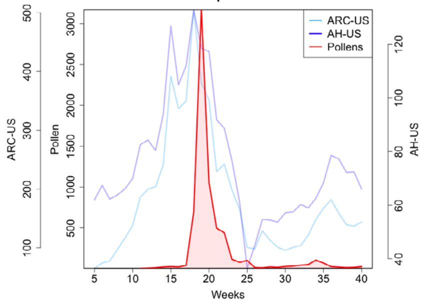

MO total pollen count

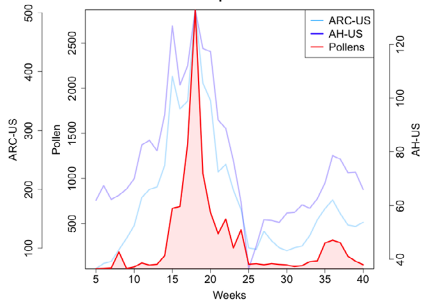

MT total pollen count

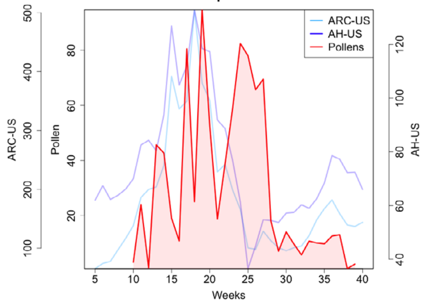

NC total pollen count

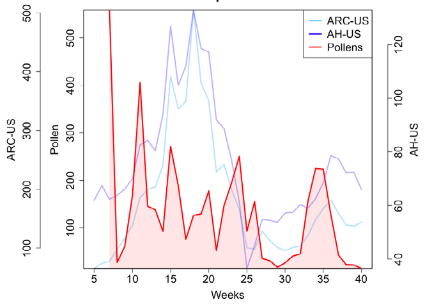

NE total pollen count

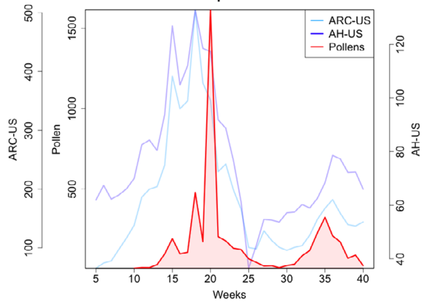

NJ total pollen count

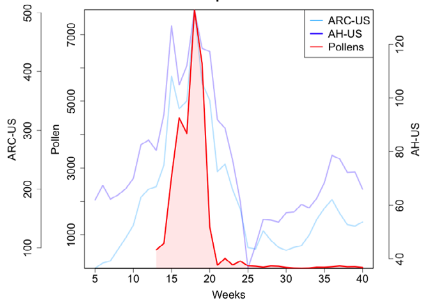

NV total pollen count

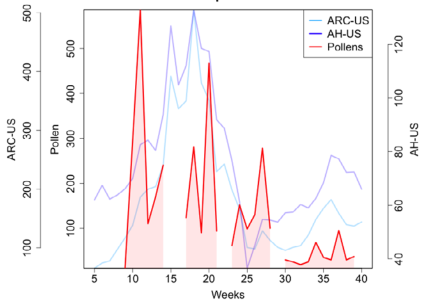

NY total pollen count

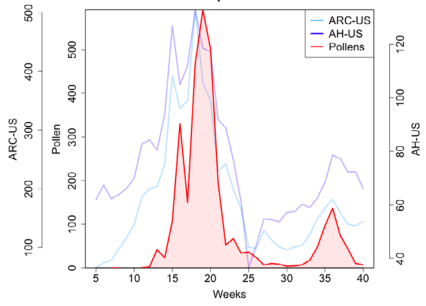

OH total pollen count

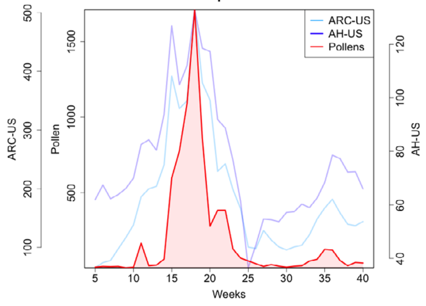

OK total pollen count

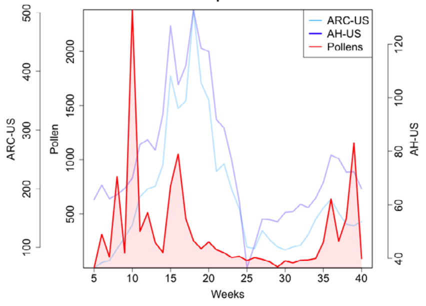

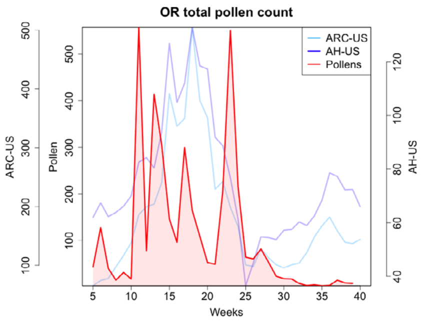
OR total pollen count
ARC-US
AH-US
Pollens
Weeks
Pollen

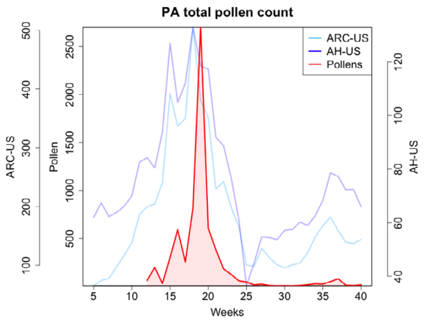
PA total pollen count
ARC-US
AH-US
Pollens
Weeks
Pollen

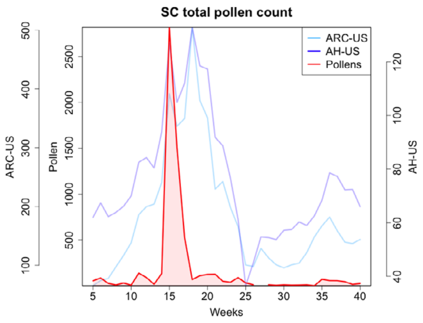
SC total pollen count
ARC-US
AH-US
Pollens
Weeks
Pollen

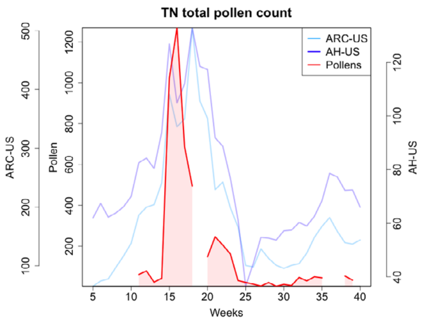
TN total pollen count
ARC-US
AH-US
Pollens
Weeks
Pollen

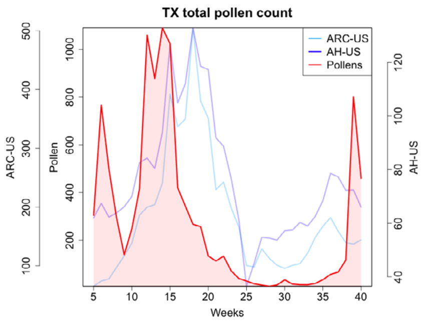
TX total pollen count
ARC-US
AH-US
Pollens
Weeks
Pollen

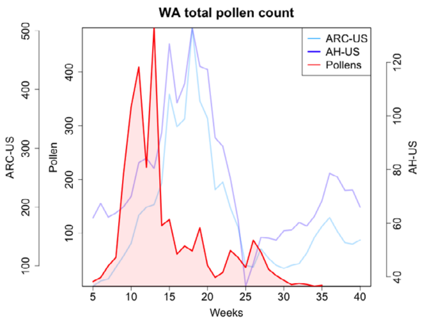
WA total pollen count
ARC-US
AH-US
Pollens
Weeks
Pollen

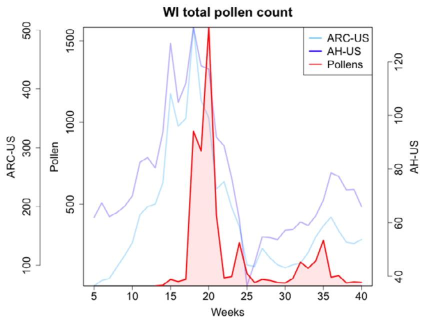
WI total pollen count
ARC-US
AH-US
Pollens
Weeks
Pollen

Pollen count trend for all cities

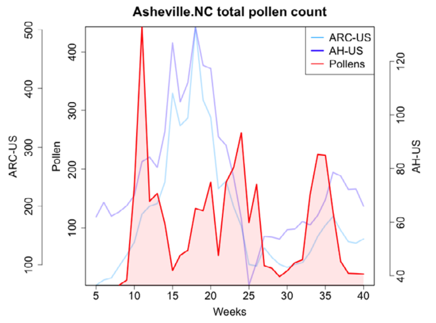

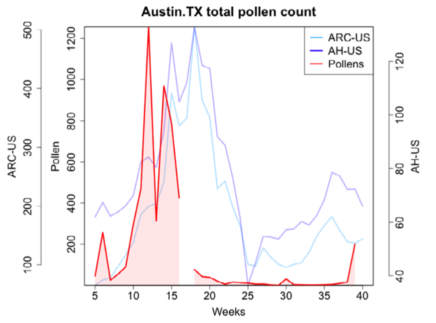

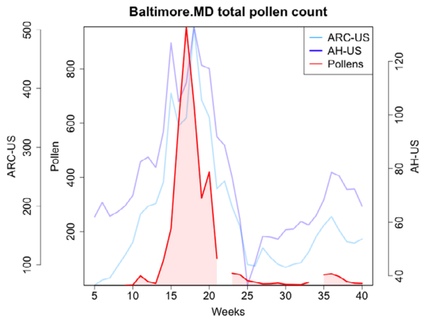

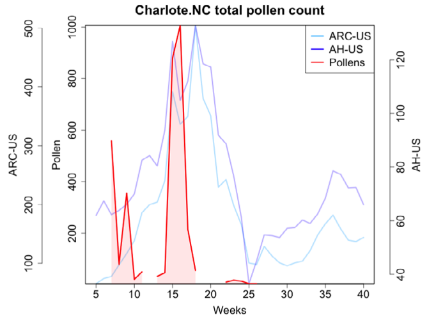

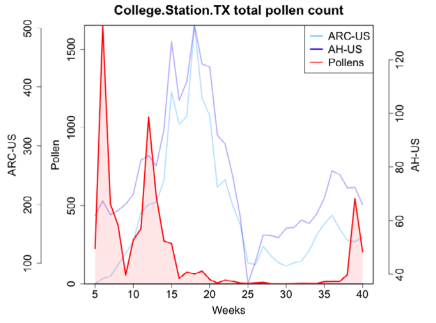

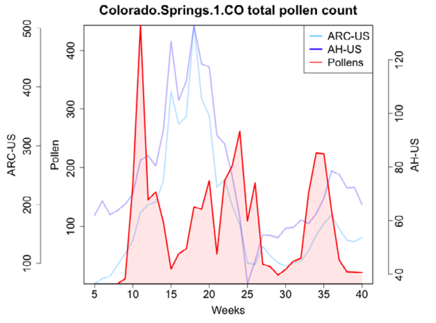

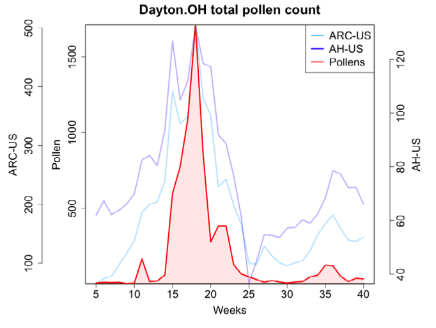

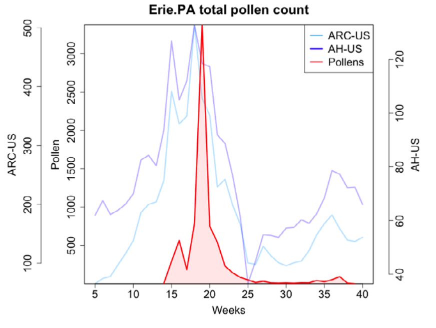

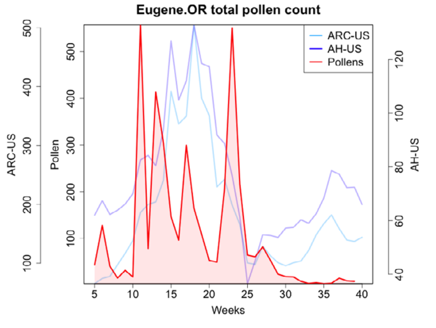

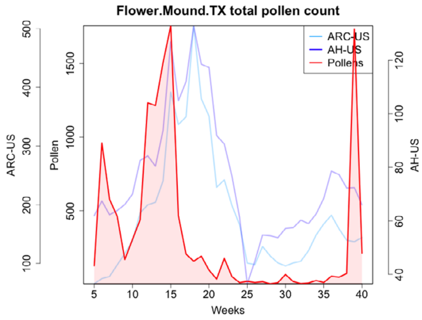
Flower.Mound.TX total pollen count
ARC-US
AH-US
Pollens
ARC-US
Pollen
AH-US
Weeks

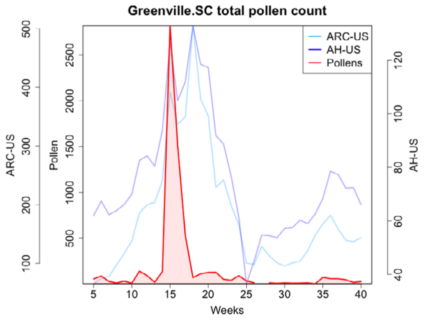
Greenville.SC total pollen count
ARC-US
AH-US
Pollens
ARC-US
Pollen
AH-US
Weeks

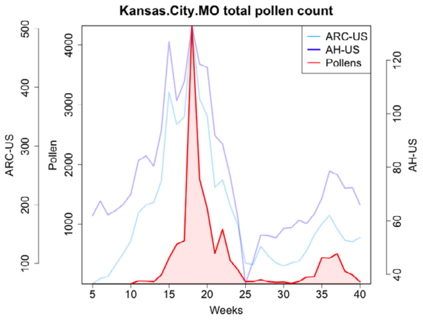
Kansas.City.MO total pollen count
ARC-US
AH-US
Pollens
ARC-US
Pollen
AH-US
Weeks

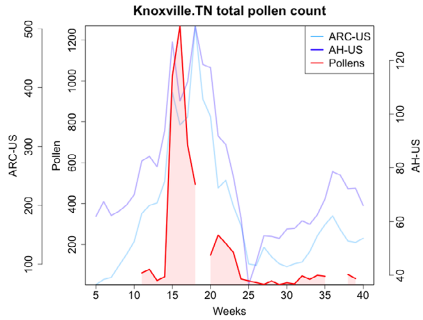
Knoxville.TN total pollen count
ARC-US
AH-US
Pollens
ARC-US
Pollen
AH-US
Weeks

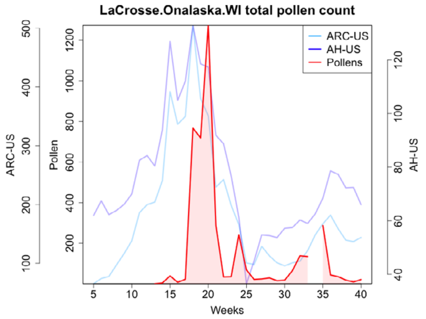
LaCrosse.Onalaska.WI total pollen count
ARC-US
AH-US
Pollens
ARC-US
Pollen
AH-US
Weeks

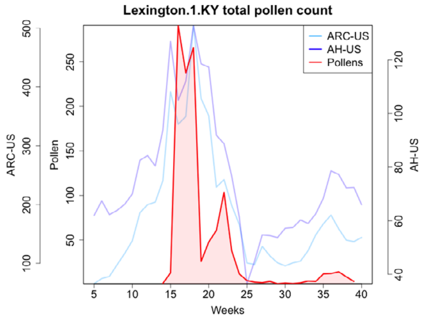
Lexington.1.KY total pollen count
ARC-US
AH-US
Pollens
ARC-US
Pollen
AH-US
Weeks

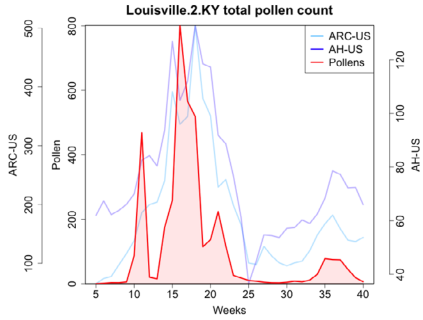
Louisville.2.KY total pollen count
ARC-US
AH-US
Pollens
ARC-US
Pollen
AH-US
Weeks

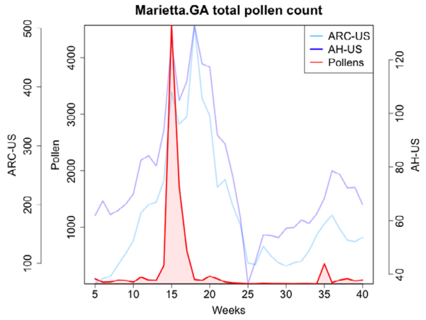
Marietta.GA total pollen count
ARC-US
AH-US
Pollens
ARC-US
Pollen
AH-US
Weeks

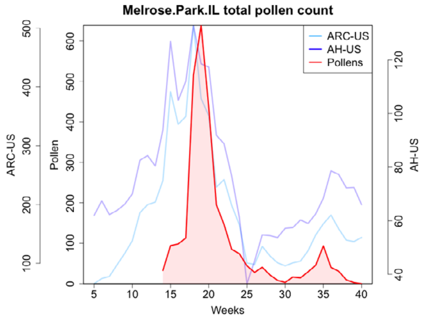
Melrose.Park.IL total pollen count
ARC-US
AH-US
Pollens
ARC-US
Pollen
AH-US
Weeks

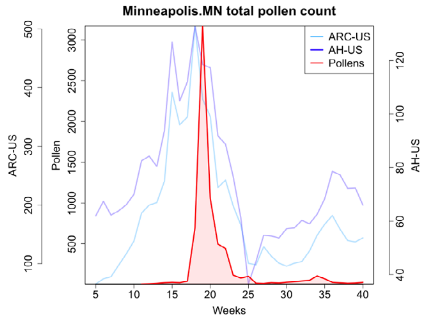
Minneapolis.MN total pollen count
ARC-US
AH-US
Pollens
ARC-US
Pollen
AH-US
Weeks

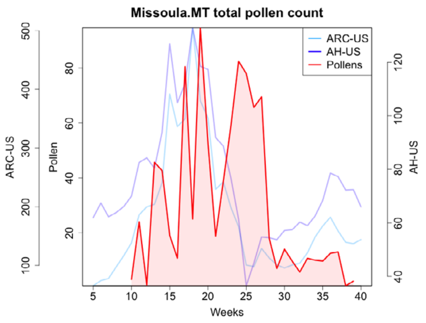
Missoula.MT total pollen count
ARC-US
AH-US
Pollens
ARC-US
Pollen
AH-US
Weeks

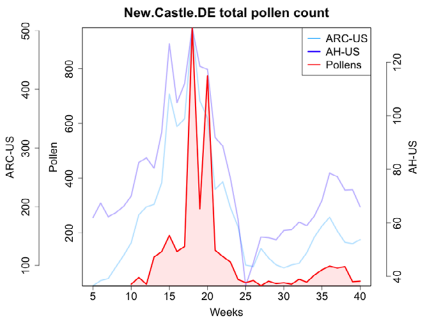
New.Castle.DE total pollen count
ARC-US
AH-US
Pollens
ARC-US
Pollen
AH-US
Weeks

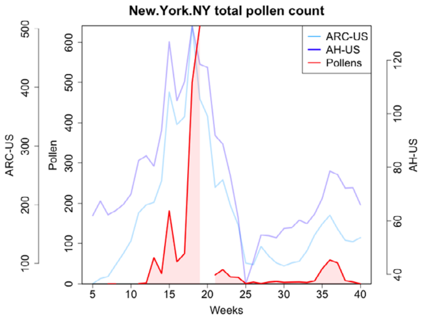
New.York.NY total pollen count
ARC-US
AH-US
Pollens
ARC-US
Pollen
AH-US
Weeks

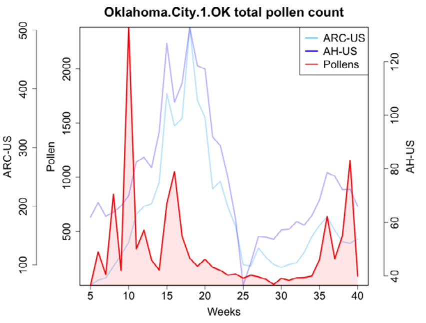
Oklahoma.City.1.OK total pollen count
ARC-US
AH-US
Pollens
ARC-US
Pollen
AH-US
Weeks

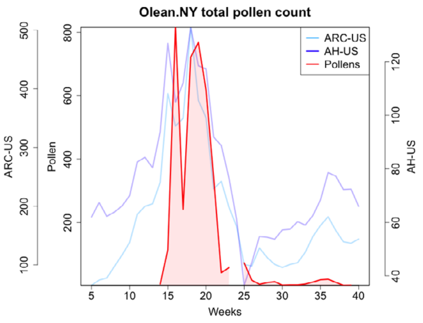
Olean.NY total pollen count
ARC-US
AH-US
Pollens
ARC-US
Pollen
AH-US
Weeks

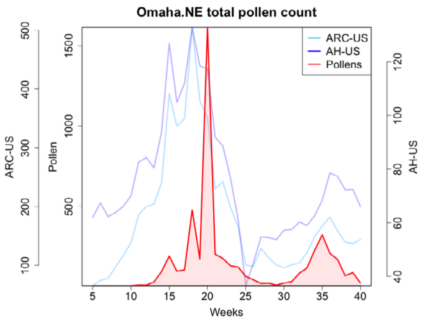
Omaha.NE total pollen count
ARC-US
AH-US
Pollens
ARC-US
Pollen
AH-US
Weeks

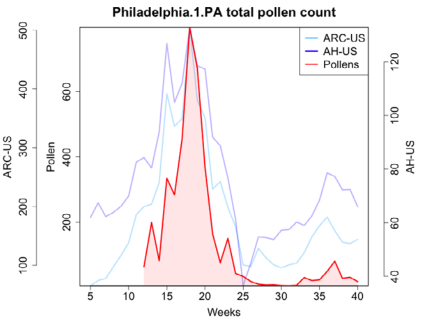
Philadelphia.1.PA total pollen count
ARC-US
AH-US
Pollens
ARC-US
Pollen
AH-US
Weeks

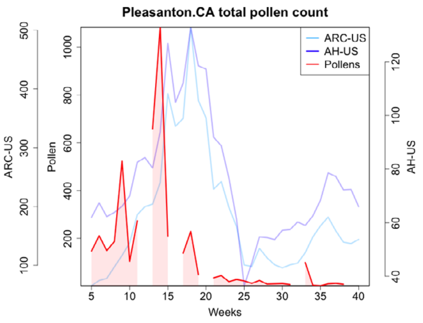
Pleasanton.CA total pollen count
ARC-US
AH-US
Pollens
ARC-US
Pollen
AH-US
Weeks

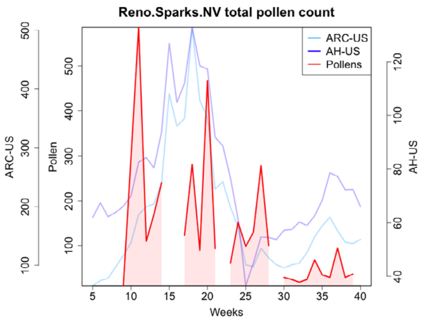
Reno.Sparks.NV total pollen count
ARC-US
AH-US
Pollens
ARC-US
Pollen
AH-US
Weeks

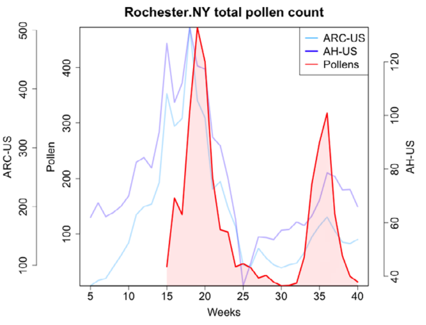
Rochester.NY total pollen count
ARC-US
AH-US
Pollens
ARC-US
Pollen
AH-US
Weeks

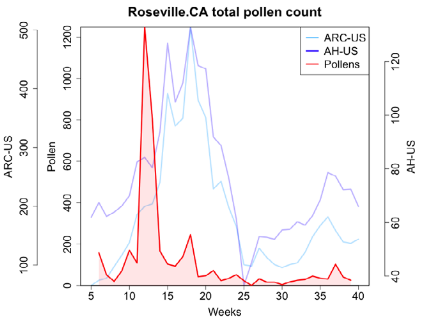
Roseville.CA total pollen count
ARC-US
AH-US
Pollens
ARC-US
Pollen
AH-US
Weeks

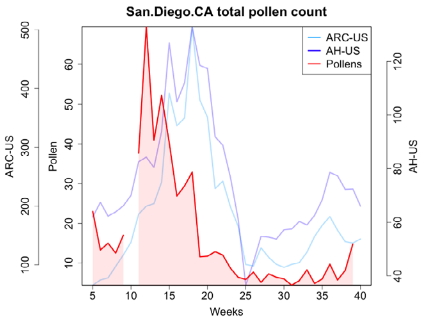
San.Diego.CA total pollen count
ARC-US
AH-US
Pollens
ARC-US
Pollen
AH-US
Weeks

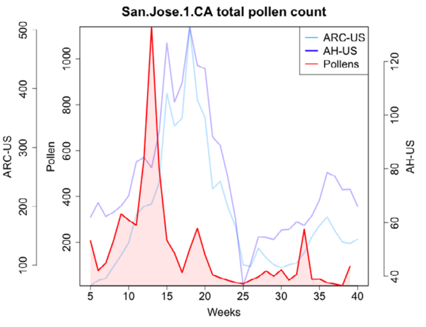
San.Jose.1.CA total pollen count
ARC-US
AH-US
Pollens
ARC-US
Pollen
AH-US
Weeks

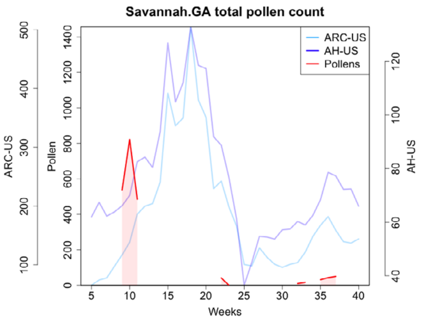
Savannah.GA total pollen count
ARC-US
AH-US
Pollens
ARC-US
Pollen
AH-US
Weeks

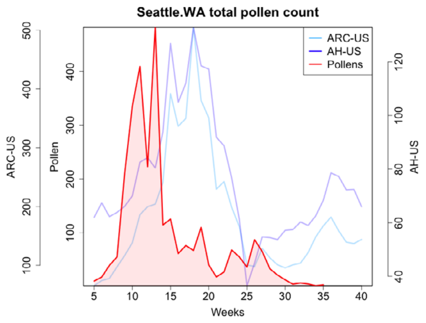
Seattle.WA total pollen count
ARC-US
AH-US
Pollens
Weeks
Pollen
ARC-US
AH-US

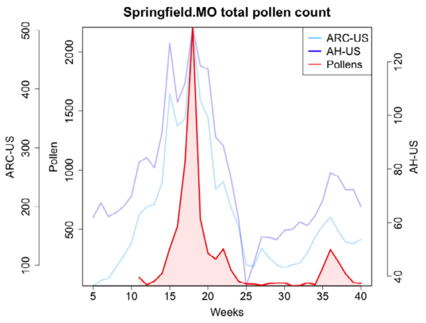
Springfield.MO total pollen count
ARC-US
AH-US
Pollens
Weeks
Pollen
ARC-US
AH-US

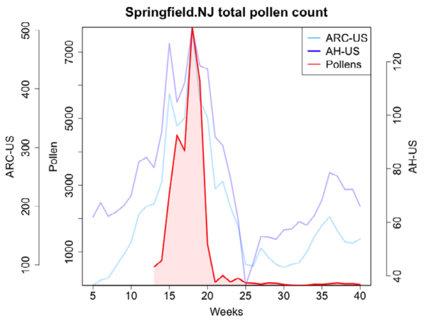
Springfield.NJ total pollen count
ARC-US
AH-US
Pollens
Weeks
Pollen
ARC-US
AH-US

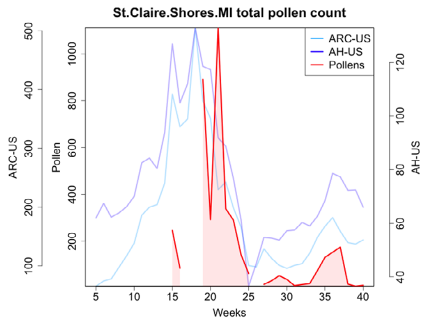
St.Claire.Shores.MI total pollen count
ARC-US
AH-US
Pollens
Weeks
Pollen
ARC-US
AH-US

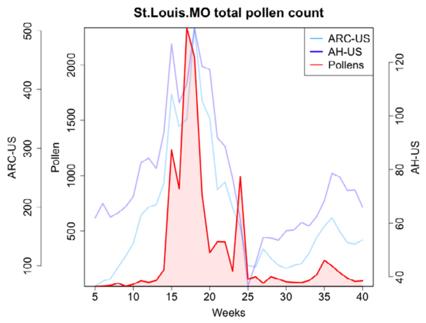
St.Louis.MO total pollen count
ARC-US
AH-US
Pollens
Weeks
Pollen
ARC-US
AH-US

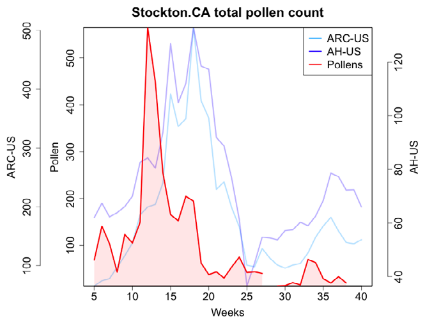
Stockton.CA total pollen count
ARC-US
AH-US
Pollens
Weeks
Pollen
ARC-US
AH-US

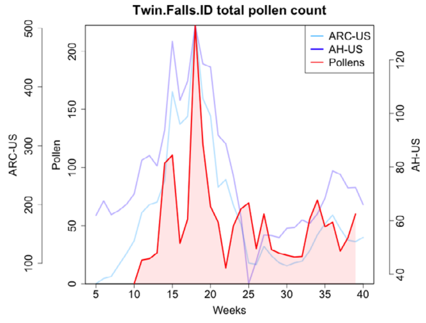
Twin.Falls.ID total pollen count
ARC-US
AH-US
Pollens
Weeks
Pollen
ARC-US
AH-US

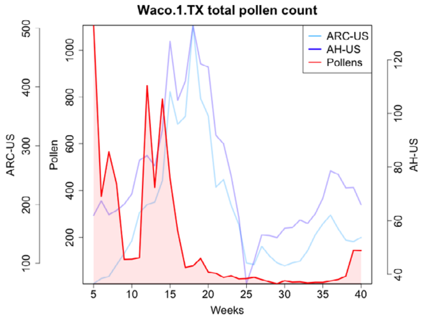
Waco.1.TX total pollen count
ARC-US
AH-US
Pollens
Weeks
Pollen
ARC-US
AH-US

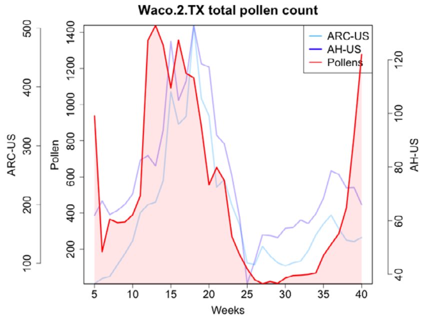
Waco.2.TX total pollen count
ARC-US
AH-US
Pollens
Weeks
Pollen
ARC-US
AH-US

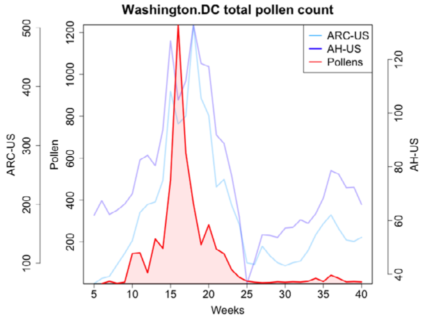
Washington.DC total pollen count
ARC-US
AH-US
Pollens
Weeks
Pollen
ARC-US
AH-US

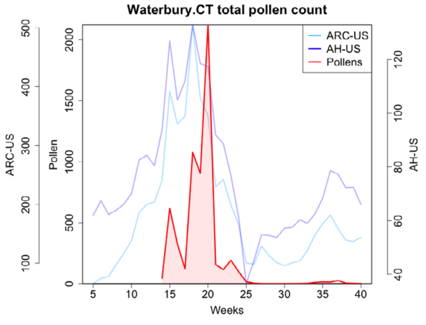
Waterbury.CT total pollen count
ARC-US
AH-US
Pollens
Weeks
Pollen
ARC-US
AH-US

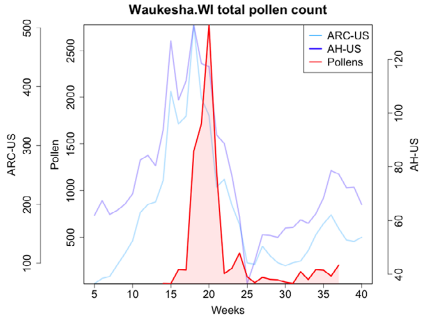
Waukesha.WI total pollen count
ARC-US
AH-US
Pollens
Weeks
Pollen
ARC-US
AH-US
